# parazitCUB: An R package to streamline the process of investigating the adaptations of parasites' codon usage bias

**DOI:** 10.12688/f1000research.143223.1

**Published:** 2023-11-02

**Authors:** Ali Mostafa Anwar, Salma Bayoumi, Sagy Elzalabany, Sameh Magdeldin, Amr E. Ahmed

**Affiliations:** 1 Biotechnology and Life Sciences Department, Faculty of Postgraduate Studies for Advanced Sciences, Beni-Suef University, Beni-Suef, Egypt; 2 Biochemistry Department, Faculty of Science, Alexandria University, Alexandria, Alexandria Governorate, Egypt; 3 Biomedical Equipment Department, Badr University in Cairo, Badr City, Cairo Governorate, Egypt; 4 Department of Physiology, Faculty of Veterinary Medicine, Suez Canal University, Ismailia, Ismailia Governorate, Egypt; 5 Basic Research Department, Children’s Cancer Hospital 57357, Cairo, Egypt, Cairo, Egypt

**Keywords:** Molecular evolution, Natural selection, Adaptation, parasites, Codon Usage Bias, R, RStudio

## Abstract

Examining the intricate association between parasites and their hosts, particularly at the codon level, assumes paramount importance in comprehending evolutionary processes and forecasting the characteristics of novel parasites. While diverse metrics and statistical analyses are available to explore codon usage bias (CUB), there presently exists no dedicated tool for examining the co-adaptation of codon usage between parasites and hosts. Therefore, we introduce the parazitCUB R package to address this challenge in a scalable and efficient manner, as it is capable of handling extensive datasets and simultaneously analyzing of multiple parasites with optimized performance. parazitCUB enables the elucidation of parasite-host interactions and the evolutionary patterns of parasites through the implementation of various indices, cluster analysis, multivariate analysis, and data visualization techniques. The tool can be accessed at the following location: https://github.com/AliYoussef96/parazitCUB

## Introduction

The transfer of genetic information from messenger RNAs (mRNAs) to proteins occurs through codons, which are sequences of three nucleotides representing amino acids. With the exception of methionine (Met) and tryptophan (Trp), most amino acids can be encoded by multiple codons, resulting in codon degeneracy. Based on studies conducted on multiple organisms, synonymous codons, which encode the same amino acid, are not uniformly utilized within genes or across different genes in the same genome, leading to codon usage bias (CUB) phenomenon. ^1^ In every organism, specific preferred (optimal) codons exist, which are utilized more frequently in highly expressed genes compared to genes with lower expression levels. ^2^ The codon usage of an organism is influenced by two major forces: mutation pressure and natural selection. Nucleotide composition, synonymous substitution rate, tRNA abundance, codon hydropathy, DNA replication initiation sites, gene length, and expression level are all known to impact the CUB. ^1^

Intracellular parasites can be categorized as facultative or obligate. Facultative parasites can reproduce both inside and outside host cells, whereas obligate parasites are unable to replicate outside their host cells and solely depend on the host cell’s resources for reproduction. ^3^ Previous studies have shown that translational selection and/or directed mutational pressure shape the codon usage of intracellular parasite genomes to optimize or deoptimize it towards the codon usage of their hosts. ^3^^,^^4^ Previous investigations have emphasized the significance of examining the interplay between parasites and the codon usage of their hosts. For instance, research conducted on the Influenza A virus (IAV) has demonstrated that understanding the patterns of codon usage in viruses might aid in the development of novel vaccines through the use of Synthetic Attenuated Virus Engineering (SAVE), which involves weakening a virus by deoptimizing its viral codons. ^5^ Similarly, another study demonstrated that the replacement of natural codons with synonymous triplets possessing higher CpG frequencies can effectively deactivate poliovirus infectivity. ^6^ To understand how parasites interact with their hosts and how they evolve, it is crucial to investigate the composition of parasite genes at the codon or nucleotide level. This analysis could assist in uncovering the mechanisms underlying parasite-host interactions and help in predicting the characteristics of newly discovered parasites.

A variety of metrics have been established to evaluate Codon Usage Bias (CUB), including the effective number of codons (ENc), codon adaptation index (CAI), relative synonymous codon usage (RSCU), and translational selection index (P2-index). ^7^ Statistical analyses, such as correspondence analysis and the Neutrality Plot, have been employed to explore the influence of selection and mutation on molding CUB. ^7^ Various tools and packages, such as coRdon, ^9^ CodonW ( http://codonw.sourceforge.net), and BCAWT. ^8^ are available for assessing and measuring CUB. However, there is currently a lack of specialized software specifically designed to examine the co-adaptation of codon usage between parasites and their hosts. The only available package developed for studying the interaction of codon usage between viruses and hosts was created in 2019 by the same first author of this research, known as vhcub R package.

The infection of multiple organisms by various parasites is a widespread phenomenon, exemplified by the existence of 1424 known viruses that can affect humans, as documented in the virus-host database. ^9^ Investigating the co-evolution of codon usage between parasites and their respective hosts presents a challenging task in the field of bioinformatics. However, thanks to modern techniques and software advancements, this endeavor has become feasible. To address this challenge in a scalable and efficient manner, the parazitCUB tool was developed. The ParazitCUB, in contrast to its predecessor vhcub package, offers an expanded scope that goes beyond virus-host interactions. It encompasses the co-evolution of codon usage between parasites and hosts, providing a more comprehensive analysis. Notably, ParazitCUB allows for the examination of larger and more extensive datasets, making it well-suited for handling substantial amounts of data. Additionally, it enables the concurrent study of multiple parasites, a feature lacking in vhcub, which only permits the analysis of a single organism with its host. Moreover, ParazitCUB has undergone significant optimization of its functions, resulting in improved speed and performance. A notable advantage of ParazitCUB lies in its user-friendly interface, facilitating effortless utilization even for users with limited proficiency in R programming.

## Methods

### Implementation

**Figure 1.**
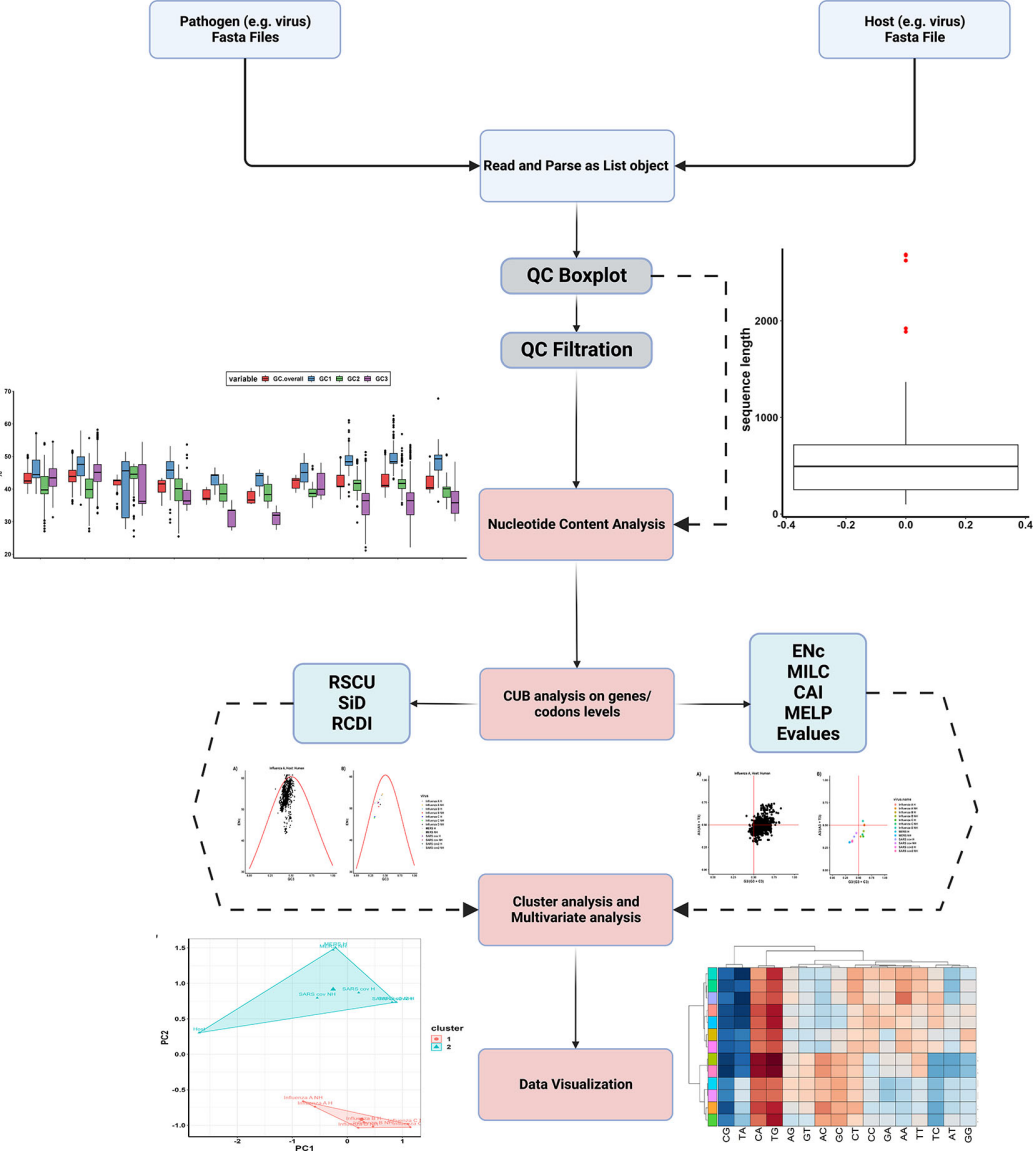
The Workflow of ParazitCUB.

ParazitCUB employs several packages, such as Biostrings, ^10^ seqinr, ^11^ and stringr, ^12^ to handle FASTA formate files and perform DNA sequence modifications. For CUB and multivariate analysis, the package utilizes coRdon and factoextra, ^13^ as well as new functions implementation. To visualize the data effectively, ParazitCUB utilizes, ggplot2, ^14^ pheatmap, ^15^ and RColorBrewer. ^16^ ParazitCUB efficiently extracts DNA sequences in FASTA format for each organism under study. These sequences are then combined into a comprehensive list. The package encompasses various indices for investigating CUB, as well as cluster analysis, multivariate analysis, and data visualization. A comprehensive list of the package’s functions, along with their corresponding results, can be found in Table 1. As well as, the package workflow has also been summarized in ( Figure 1).

**Table 1.** A comprehensive list of the package’s functions, along with their corresponding results.

Function name	Description	Value
fasta.files	Read FASTA files for parasites and combine them in a list with file name as the name of the organism	A list containing Biostrings objects
read.host	Read FASTA file of a organism	A list containing Biostrings objects
GC.content	Calculates overall GC content as well as GC at first, second, and third codon positions.	A list containing data.frames with GC content at first, second, and third codon positions
ENc.values.old	Measure the Effective Number of Codons (ENc)	A list of data.frames containing the computed ENc
ENc.values.new	Measure the Effective Number of Codons (ENc), using its modified version	A list of data.frames containing the computed modified ENc
MILC.values	Measure the Independent of Length and Composition	A list of data.frames containing the computed MILC
B.values	Measure the codon bias, termed Band A list of data.frames containing the computed B index	A list of data.frames containing the computed B Values
MCB.values	Measure the maximum likelihood codon bias	A list of data.frames containing the computed MCB
CAI.values	Measure the Codon Adaptation Index (CAI) using	A list of data.frames containing the computed CAI
MELP.values	Measure the MILC-based Expression Level Predictor	A list of data.frames containing the computed MELP
FOP.values	Measure the frequency of optimal codons	A list of data.frames containing the computed FOP
E.values	Measure the related measure of expression	A list of data.frames containing the computed E index
RSCU.values	Measure the Relative Synonymous Codon Usage	A list of data.frames containing the computed RSCU
SiD.list	Measure the Similarity Index (SiD) between a parasite and its host codon usage	A list of data.frames containing the computed SiD
RCDI.calc	Measure the Relative Codon Deoptimization Index	A list of data.frames containing the computed RCDI
dinuc.base	A measure of statistical dinucleotide over- and under-representation; by allows for random sequence generation by shuffling (with/without replacement) of all bases in the sequence	A list of data.frames containing the computed statistic for each dinucleotide
dinuc.codon	A measure of statistical dinucleotide over- and underrepresentation; by allows for random sequence generation by shuffling (with/without replacement) of codons	A list of data.frames containing the computed statistic for each dinucleotide
dinuc.syncodon	A measure of statistical dinucleotide over- and underrepresentation; by allows for random sequence generation by shuffling (with/without replacement) of synonymous codons	A list of data.frames containing the computed statistic for each dinucleotide
QC.cutoff	Remove Coding sequences with minimum and maximum number of amino acids	A list containing Biostrings objects
QC.boxplot	Plot the number of Coding sequences amino acids count as QC plot	A ggplot object
GC.boxplot	Make a box plots for GC content	A ggplot object
ENc.GC3plot	Make an ENc-GC3 scatterplot.	A ggplot object
ENc.GC3plot.group	Make a group ENc-GC3 scatterplot.	A ggplot object
PR2.plot	Make a Parity rule 2 (PR2) plot	A ggplot object
PR2.plot.group	Make a group Parity rule 2 (PR2) plot	A ggplot object
Neutrality.plot	Make a Neutrality plot	A ggplot object
cub.heatmap	RSCU or dinucleotide Heatmap	A ggplot object
rscu.pca	Principal component analysis using RSCU values	A ggplot object
rscu.cluster	Cluster analysis on PCA of RSCU values	A ggplot object

### Operation

parazitCUB was developed in R, and the source code can be found on GitHub and archived with Zenodo. ^20^ It works with Windows and most Linux operating systems.

1 # install devtools if it is not available
2 # install.packages("devtools")
3 devtools::install_github("AliYoussef96/parazitCUB")


The parazitCUB package consists of six main branches, each serving a distinct purpose: nucleotide content analysis, CUB analysis at the gene level, CUB analysis at the codon level, cluster analysis, multivariate analysis, and data visualization. Within each branch, a range of methods is available for conducting CUB studies. The complete workflow of ParazitCUB is illustrated in Figure 1, providing an overview of the entire process. For comprehensive information on using ParazitCUB, detailed documentation is readily available https://github.com/AliYoussef96/parazitCUB.

## Use cases

The utilization of parazitCUB for investigating codon usage bias (CUB) in viruses (or any type of parasites), their respective hosts, and the co-adaptation between them offers a straightforward and highly customizable approach. To exemplify the capabilities of the package, the coding sequences of seven viruses, namely Influenza A, Influenza B, Influenza C, Influenza D, MERS, SARS-CoV, and SARS-CoV-2, were obtained from NCBI virus gateway. ^17^ To showcase the package’s ability to handle larger datasets, two variants of each virus were downloaded, with one variant isolated from a non-human host and the other from a human host (except for Influenza D).

To begin, all the fasta files for the viruses should be located in a single directory, such as a folder named “virus fasta” To read all the files simultaneously for parazitCUB analysis, the following straightforward approach can be employed:

1 library("parazitCUB")
2 library("ggplot2")
3 fasta.files <- list.files("flu fasta/", pattern = ".fasta", full.names = T)
4 list.virus <- read.virus (fasta.files, sep = "|")
5 # The sep parameter to mange long headers in fasta files.


After importing the host FASTA file, we focused exclusively on the human host for this particular analysis (only one host selection is permitted). In this instance, the genes exhibiting the highest expression levels in human lung tissues were collected from the Human Protein Atlas project database.

1 theHost <- read.host("human fasta/Human.fasta", sep = "|")


A reasonable quality control step is to examine the coding sequence length in the virus datasets, to remove any bias (very long sequences, or very short ones) that could negatively affect the result. parazitCUB provides an easy straightforward function to do that.

1 QC.boxplot (list.virus)


**Figure 2.**
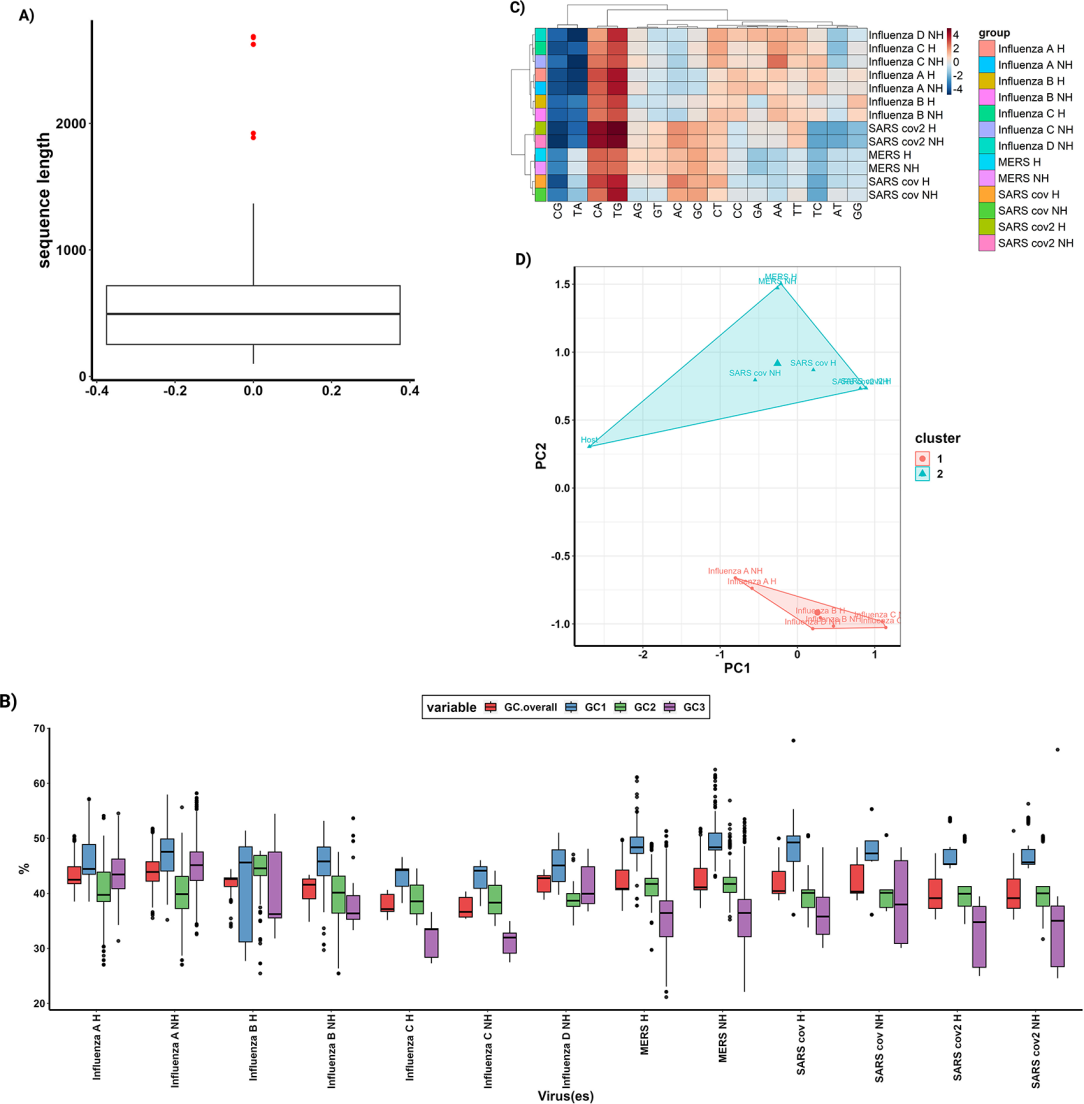
“Quality Control,” “Nucleotide Content,” “Cluster Analysis,” and “Multivariate Analysis” within the ParazitCUB package. A) A box plot for the lengths of all coding sequences in the study, serving as a quality control measure. With outliers displayed as red dots. B) A box plot illustrates the GC content of all organisms in the study for each codon position and provides an overall view of the GC content. C) A heatmap, combined with cluster analysis, utilizes the statistical representation of dinucleotide over- and underrepresentation to visually depict patterns and similarities among the data. D) Conducting cluster analysis on a Principal Component Analysis (PCA) using the RSCU values for each organism in the study enables the identification of clusters and relationships based on codon usage.

This function will create a boxplot ( Figure 2A) which illustrates the distribution of coding sequence lengths across the study. Through the examination of outliers in the boxplot, the QC.cutoff() function can be employed to exclude extremely long and short sequences from subsequent analyses, thereby enhancing the integrity of the data.

1 list.virus <- QC.cutoff (list.virus, cut.off.up = 4000, cut.off.down = 100)


To exemplify the provided CUB workflow; we will show a case study involving the utilization of various functions from each of the six branches of the package.

### Nucleotide content analysis

To compute the GC content at every position across all viruses included in the study:

1 GC.list <- GC.content (list.virus)


GC.boxplot() function, which produces a graphical representation of the GC content distribution ( Figure 2B).

1 GC.boxplot (GC.list) + theme (axis.text.x = element_text(angle = 90, vjust = 0.5, hjust =1))


All visualizations within the parazitCUB package are created using ggplot2, which allows for convenient customization of the default plot parameters.

Furthermore, the package provides the capability to calculate the statistical over- and underrepresentation of dinucleotides using different models such as base, codon, and synonymous codons. The calculation can be performed as follows:

1 base <- dinuc.base (list.virus, permutations = 100)
2 codon <- dinuc.codon (list.virus, permutations = 100)
3 syncodon <- dinuc.syncodon (list.virus, permutations = 100)


### CUB analysis on genes/codons levels

As part of this section, numerous indices can be calculated to assess Codon Usage Bias (CUB). For example, the effective number of codons (ENc) can be determined using the ENc.values.new() function, which utilizes a modified version. ^18^ Also, MILC.values(), B.values(), and MCB.values() functions could be used to calculate the MILC, B, and MCB, respectively. All of these functions can work on the virus coding sequence without the need for a host coding sequence as a reference set.

1 enc.list <- ENc.values.new (list.virus)


Additionally, the MILC.values(), B.values(), and MCB.values() functions can be employed to calculate the MILC, B, and MCB indices, respectively. It is important to note that these functions can operate on the virus coding sequence alone, without requiring a host coding sequence as a reference set.

1 MILC.list.virus <- MILC.values (list.virus)


Some indices within the ParazitCUB package require a reference gene set to ensure their accurate computation and cannot be executed without it. One such example is:

1 MILC.list.virus <- MILC.values (list.virus, host = theHost)


Certain indices rely on a reference genes set for their proper functioning and cannot operate without it. For instance;

1 cai.list <- CAI.values (list.virus, host = theHost) # To calculate the Codon Adaptation Index.
2 melp.list <- MELP.values (list.virus, host = theHost) # To calculate the MILC – based Expression Level Predictor.
3 E.values <- E.values (list.virus, host = theHost) # To calculate the Related measure of expression.


Moreover, various matrices are provided within ParazitCUB to facilitate the examination of Codon Usage Bias (CUB) at the codon level. For instance:

1 rscu.virus <- RSCU.values (list.virus) # To calculate the Relative synonymous codon usage. Could be used for the virus and the host.
2 rscu.host <- RSCU.values (theHost)
3 SiD <- SiD.list (RSCU.host = rscu.host, RSCU.virus = rscu.virus) # To calculate similarity index between the RSCU of the virus and the host.
4 rcdi <- RCDI.calc (list.virus, theHost, rscu.host, enc. host) # To calculate Relative codon deoptimization index.


### Cluster analysis and Multivariate analysis

Cluster analysis and multivariate analysis have been widely utilized in numerous research studies to explore codon usage patterns. Within the framework of parazitCUB, three essential functions have been integrated for this purpose. One of these functions, cub.heatmap(), facilitates the generation of a heatmap using either Relative Synonymous Codon Usage (RSCU) values or statistical representations of dinucleotide over- and underrepresentation. Additionally, cub.heatmap() supports the utilization of various clustering methods implemented through the R stats function hclust() ^19^ ( Figure 2C).

1 codon <- dinuc.codon (list.virus, permutations = 10)
2 cub.heatmap (codon, cluster_rows = T, aver = T, clustering_distance_rows = "euclidean", clustering_distance_cols = "euclidean", 
3      clustering_method = "ward. D")


Principal Component Analysis (PCA) is a commonly employed technique to reduce the dimensionality of RSCU values and identify the primary sources of variation and factors influencing codon usage within an organism. This task can be easily performed using the parazitCUB package ( Figure 2D).

1 rscu.pca (rscu.virus, rscu.host, codons.exclude = c("ATG", "TAA", "TAG", "TGA", "TGG"))


Subsequently, cluster analysis can be conducted based on the PCA results as follows:

1 rscu.cluster (rscu.virus, rscu.host, k = 2, rank = 2, FUNcluster = "kmeans",
2       hc_metric = "euclidean", hc_method = "ward.D2")


### Data visualization

**Figure 3.**
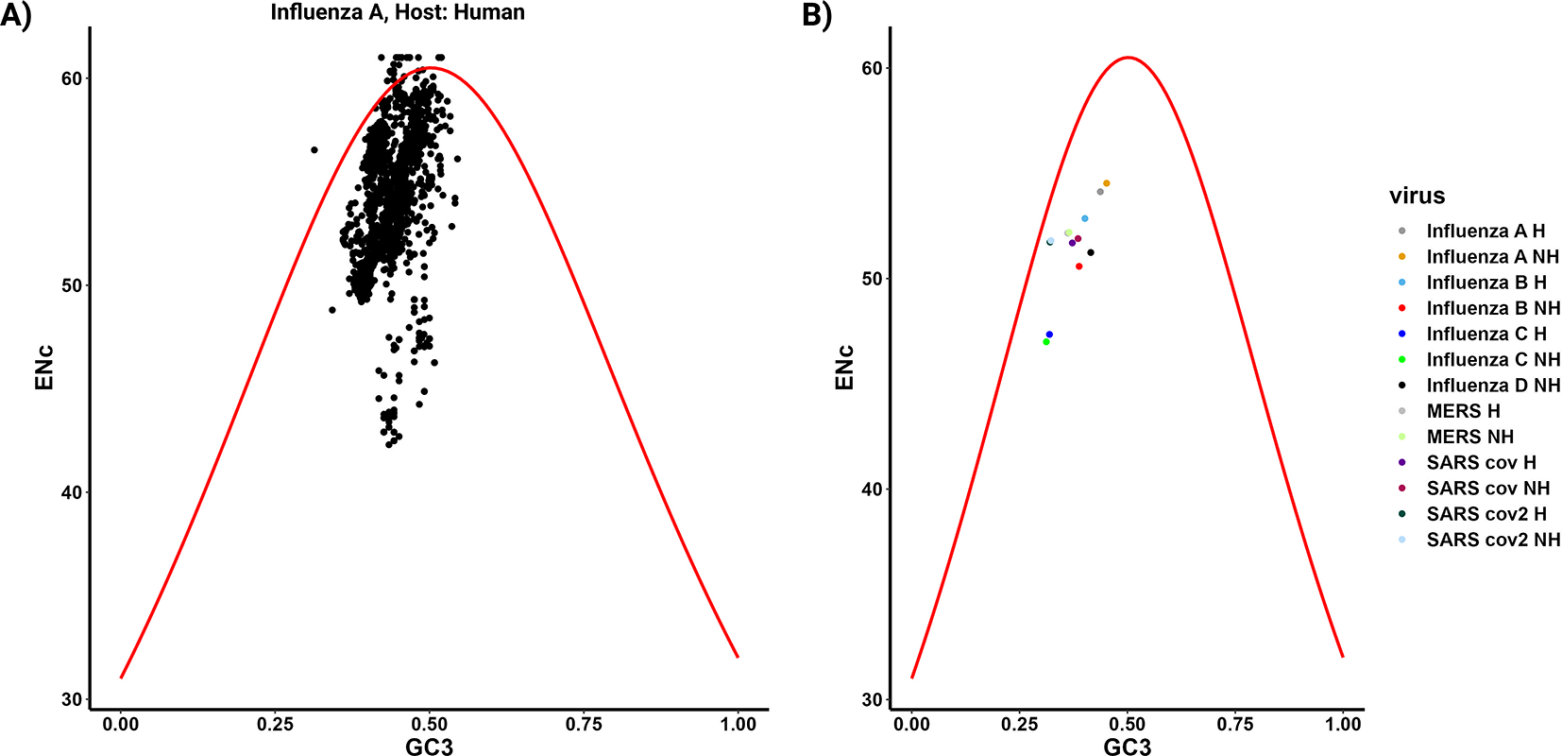
ENc-GC3 analysis implemented in parazitCUB. A) ENc-GC3 plot displays the ENc values plotted against the GC3 content for the virus Influenza A (human CDS as reference) CDS. In this plot, the solid red line represents the expected ENc values when the codon bias is solely influenced by GC3s. B) The plot represents the average effect of ENc-GC3 for all organisms included in the study.

**Figure 4.**
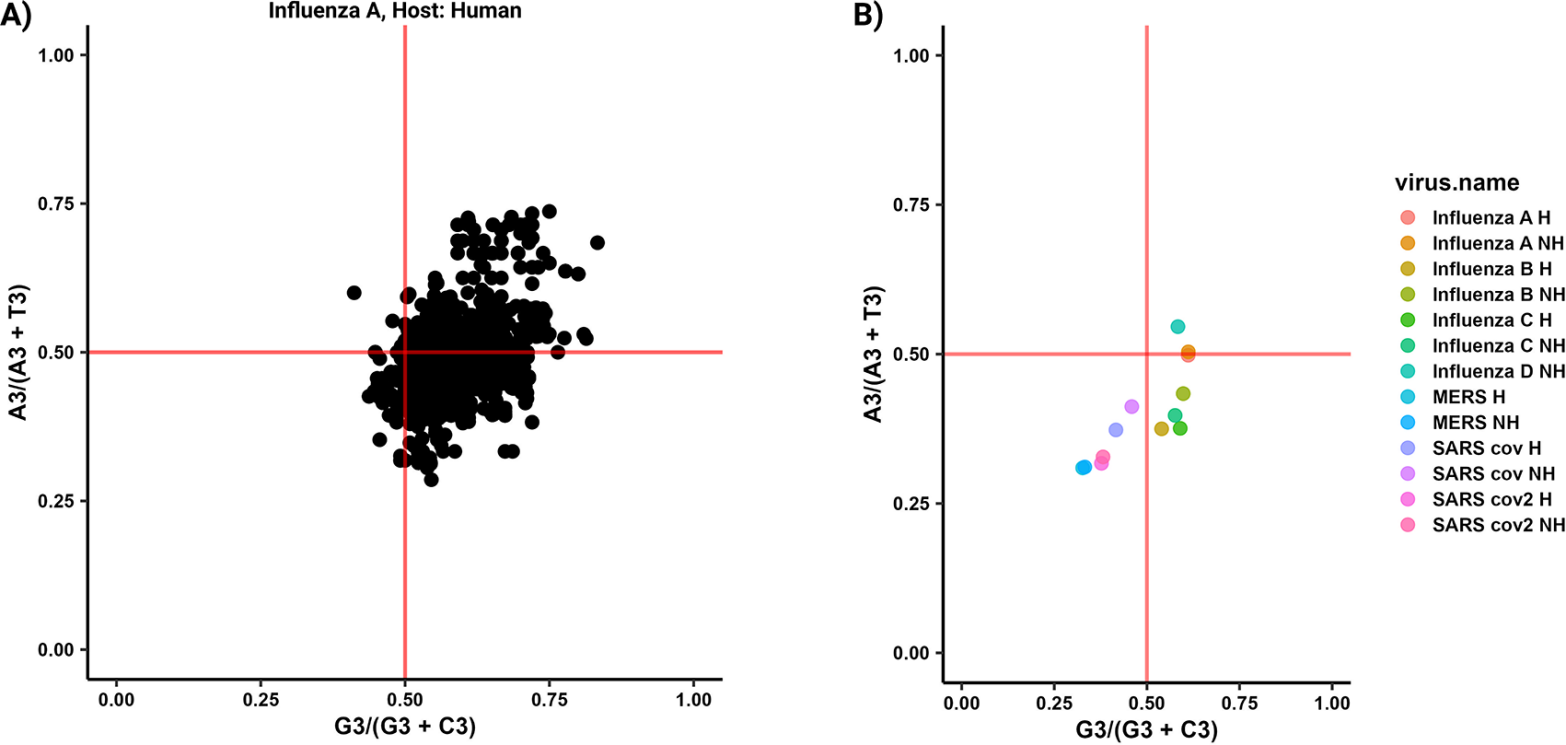
PR2-plot analysis implemented in parazitCUB. A) A PR2-plot illustrates the coding sequences (CDS) of the Influenza A (human CDS as reference) CDS, depicting their GC bias (ratio of G3 to G3 + C3) and AT bias (ratio of A3 to A3 + T3) in the third position of each codon. The two solid red lines on the graph indicate the point where both the vertical and horizontal coordinates are 0.5, representing the condition where A is equal to T and G is equal to C. B) The plot represents the average effect of PR2 values for all organisms included in the study.

**Figure 5.**
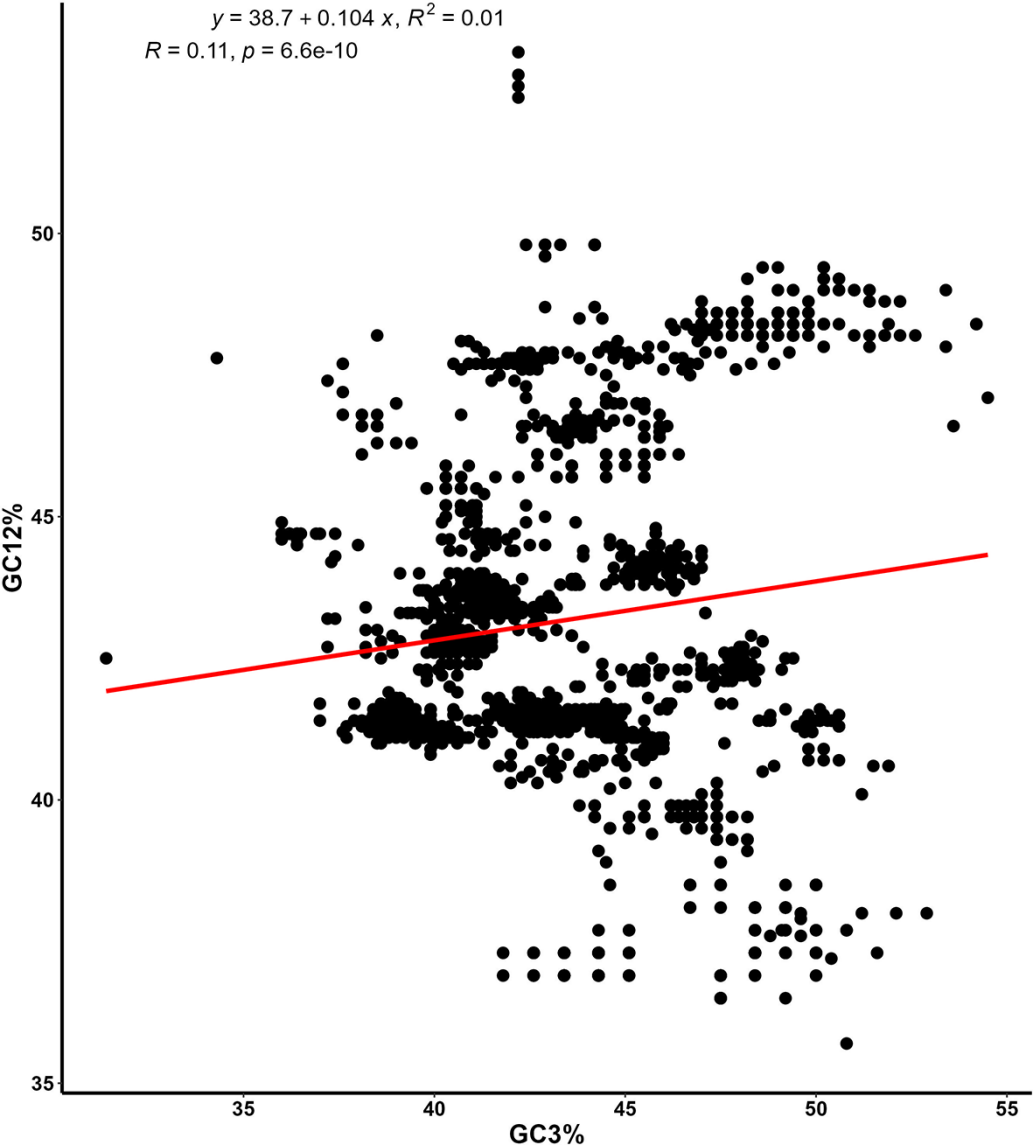
The Neutrality plot involves analyzing the GC12 and GC3 contents by plotting their frequencies against each other. On the plot, the y-axis represents the average GC frequency at the first and second codon positions (GC12), while the x-axis represents the GC frequency at the third codon position (GC3). The equation for the slope, along with the coefficient of determination (R) and its associated p-value, are shown.

The forces that impact codon usage bias (CUB), such as mutational pressure and natural selection, have been extensively explored using various plots including the ENc-GC3 plot, PR2 plot, and Neutrality plot. In parazitCUB, two versions of the ENc-GC3 plot are available: the first version displays the ENc-GC3 of a specific virus analyzed ( Figure 3A), while the second version presents the average ENc-GC3 for all the organisms studied in a single figure ( Figure 3B). The same applies to the PR2 plot ( Figure 4A and B). The Neutrality plot, can only be used for one organism at a time ( Figure 5).

1 ENc.GC3plot(enc.list[["Influenza A H"]], GC.list[["Influenza A H"]])
2 ENc.GC3plot.group (enc.list, GC.list)
3
4 
5 PR2.plot (list.virus[[1]], fold4 = TRUE)
6 PR2.plot.group (list.virus)
7
8 Neutrality.plot (GC.list[["Influenza A H"]])


## Conclusions

parazitCUB streamlines the process of investigating the adaptations of parasites’ Codon Usage Bias (CUB) within the R environment, ensuring scalability and efficiency. By allowing the study of several parasites concurrently in connection to a specific host, ParazitCUB facilitates a thorough understanding of the factors influencing parasite evolution and offers potential insights for establishing effective treatment strategies

## Data Availability

Zenodo: AliYoussef96/parazitCUB: V1.0.0. https://doi.org/10.5281/zenodo.8393578. ^20^ This project contains the following underlying data:
• Fasta Files: A folder containing all the fasta files used in the case study https://github.com/AliYoussef96/parazitCUB/tree/main/flu%20fasta

## Software availability

• Source code available from: https://github.com/AliYoussef96/parazitCUB
• Archived source code at time of publication: https://doi.org/10.5281/zenodo.8393578 ^20^
• License: GPL-3
