# Supplementary material for: parazitCUB: An R package to streamline the process of investigating the adaptations of parasites' codon usage bias
Source: F1000Res. 2023 Nov 2;12:1431. [Version 1] doi: 10.12688/f1000research.143223.1 (PMC10682597; doi:10.12688/f1000research.143223.1)
Supplement: Peer review history

# Reviewer report: Reviewer response for version 1

**DOI:** 10.5256/f1000research.156860.r220457

**Published:** 2023-11-27

**Authors:** Xianzhao Kan

**Affiliations:** 1 The Institute of Bioinformatics, College of Life Sciences, Anhui Normal University, Wuhu, China

General comments:

 parazitCUB is a useful R package for investigating codon usage co-adaptation between parasites and their hosts. Unlike its predecessor vhcub which focuses solely on virus-host interactions, ParazitCUB provides an enhanced scope. The package provides comprehensive statistical analyses, including Neutrality plot, Maximum likelihood codon bias, CU Heatmap using RSCU, and so on. Overall, I believe that ParazitCUB provides convenience for in-depth research on parasite-host co-evolution.

 Improved:

1. “Host (e.g., virus) Fasta File” in the upper right corner of Figure 1 should be “Host (e.g., human) Fasta File”.
2. To avoid repeating the word "previous," the sentence (Second paragraph of Introduction) "Previous investigations have emphasized the significance of examining the interplay between parasites and the codon usage of their hosts" should be rephrased as "This has emphasized the significance of examining the interplay between parasites and the codon usage of their hosts."

Are the conclusions about the tool and its performance adequately supported by the findings presented in the article?

Yes

Is the rationale for developing the new software tool clearly explained?

Yes

Is the description of the software tool technically sound?

Yes

Are sufficient details of the code, methods and analysis (if applicable) provided to allow replication of the software development and its use by others?

Yes

Is sufficient information provided to allow interpretation of the expected output datasets and any results generated using the tool?

Yes

Reviewer Expertise:

Molecular evolution, bioinformatics, molecular biology.

I confirm that I have read this submission and believe that I have an appropriate level of expertise to confirm that it is of an acceptable scientific standard.

# Reviewer report: Reviewer response for version 1

**DOI:** 10.5256/f1000research.156860.r220456

**Published:** 2023-11-24

**Authors:** Ahmed Abdelmonem Hemedan

**Affiliations:** 1 Luxembourg Centre for Systems Biomedicine, Luxembourg University, Luxembourg City, Luxembourg District, Luxembourg

# parazitCUB manuscript review

 The article introducing parazitCUB, an R package for analysing codon usage bias in parasites and hosts - It is a valuable addition to the field, addressing a unique research need.

 The introduction would require incorporating specific examples where codon usage bias analysis has impacted our understanding of parasitic diseases and treatment strategies.

 A thorough comparative analysis with existing tools like CodonW or coRdon is required - perhaps you would like to focus on unique features, user interface, data handling capabilities, and specific functionalities, would provide valuable insights.

 Expanding on the computational methodologies and algorithms used in parazitCUB is crucial for scientific rigor and reproducibility.

 Including a robust performance assessment section, exploring aspects like accuracy, computational efficiency, and scalability through comparative analyses, is essential.

 Detailed case studies in the use case section, demonstrating the tool's utility with specific data and analytical processes, would illustrate its practical value.

 Enhanced documentation, including a comprehensive user guide, example datasets, troubleshooting tips, and FAQs, is necessary to make the tool approachable to a broader audience.

 Enriching the paper with more effective visuals and data visualizations would aid in conveying complex data and analyses in an engaging manner.

 The discussion section should contextualise parazitCUB within the broader bioinformatics field and its potential impact on future research, including possible expansions or updates of the tool.

Are the conclusions about the tool and its performance adequately supported by the findings presented in the article?

Yes

Is the rationale for developing the new software tool clearly explained?

Partly

Is the description of the software tool technically sound?

Yes

Are sufficient details of the code, methods and analysis (if applicable) provided to allow replication of the software development and its use by others?

Yes

Is sufficient information provided to allow interpretation of the expected output datasets and any results generated using the tool?

Yes

Reviewer Expertise:

Systems medicine, Disease dynamic modelling, Biostatistics, Mathematics, Bioinformatics, Molecular Pathology.

I confirm that I have read this submission and believe that I have an appropriate level of expertise to confirm that it is of an acceptable scientific standard.
